# Effectiveness and reliability of hypnosis in stereotaxy: a randomized study

**DOI:** 10.1007/s00701-024-05943-0

**Published:** 2024-02-27

**Authors:** Sabina Catalano Chiuvé, Shahan Momjian, Adriana Wolff, Marco Vincenzo Corniola

**Affiliations:** 1 Neurology Department, Neuropsychology Unit, Geneva University Hospitals, Geneva, Switzerland; 2 Faculty of Medicine, Université of Genève, Geneva, Suisse; 3 Neurosurgery Department, Geneva University Hospitals, Geneva, Switzerland; 4 Anesthesiology Department, Geneva University Hospitals, Geneva, Switzerland

**Keywords:** Parkinson’s disease, Pain, Hypnosis, Deep brain stimulation

## Abstract

**Background:**

Patients suffering from Parkinson’s disease (PD) may experience pain during stereotactic frame (SF) fixation in deep brain stimulation (DBS). We assessed the role of hypnosis during the SF fixation in PD patients undergoing awake bilateral subthalamic nucleus (STN) DBS.

**Methods:**

*N* = 19 patients were included (*N* = 13 males, mean age 63 years; *N* = 10 allocated to the hypnosis and *N* = 9 allocated to the control groups). Patients were randomly assigned to the interventional (hypnosis and local anesthesia) or non-interventional (local anesthesia only) groups. The primary outcome was the pain perceived (the visual analogue scale (VAS)). Secondary outcomes were stress, anxiety, and depression, as measured by the perceived stress scale (PSS) and hospital anxiety and depression scale (HADS). Procedural distress was measured using the peritraumatic distress inventory (PDI-13).

**Results:**

In the hypnosis group, VAS_mean_ was 5.6 ± 2.1, versus 6.4 ± 1.2 in the control group (*p* = 0.31). Intervention and control groups reported similar VAS_max_ scores (7.6 ± 2.1 versus 8.6 ± 1.6 (*p* = 0.28), respectively). Both groups had similar HADS scores (6.2 ± 4.3 versus 6.7 ± 1.92, *p* = 0.72 (HADSa) and 6.7 ± 4.2 versus 7.7 ± 3, *p* = 0.58 (HADSd)), so were the PSS scores (26.1 ± 6.3 versus 25.1 ± 7, *p* = 0.75). Evolutions of VAS_mean_ (*R*^2^ = 0.93, 95% CI [0.2245, 1.825], *p* = 0.03) and PDI-13 scores (*R*^2^ = 0.94, 95% CI [1.006, 6.279], *p* = 0.02) significantly differ over follow-up with patients in the hypnosis groups showing lower scores.

**Conclusion:**

In this unblinded, randomized study, hypnosis does not influence pain, anxiety, and distress during awake SF fixation but modulates pain memory over time and may prevent the integration of awake painful procedures as a bad experience into the autobiographical memory of patients suffering from PD. A randomized controlled study with more data is necessary to confirm our findings.

## Introduction

The use of a stereotactic frame (SF) mounted on the patient’s head is required in deep brain stimulation (DBS) surgery. When it comes to awake procedures, the patient is able to sit up with the head straight, ensuring speedy and accurate mounting of the device. While the mounting may be reported to be painful and uncomfortable, it has to be carried out with special attention as the SF must be positioned as symmetrical as possible and parallel to Reid’s baseline [3]. In the case of awake DBS, pain and discomfort may be experienced by patients suffering from Parkinson’s disease (PD) during SF fixation as well as during scalp incision and may compromise their adherence to the procedure.

On the surgeon’s side, causing an unpleasant experience to the patient may feel distressing, discouraging, and somewhat disappointing. This can be even more true in patients suffering from PD, as it has been reported that they show lower tolerance and increased vulnerability to pain [39, 61] as well as a higher incidence of anxiety [23]. Furthermore, the procedure is mostly carried out with the patient being OFF-medication with motor and non-motor symptoms [45]. Altogether, these factors may exacerbate the perceived pain and aggravate the anxiety related to the SF mounting and the overall DBS procedure. In order to reduce pain during SF mounting on the head, local anesthesia (LA) is traditionally used at the site where the pins penetrate the skin prior to the scalp incision [43]. Alternatively, a supra-orbital block or a cranial block can be performed [59]. However, the pressure felt during pins insertion may still be perceived as uncomfortable and distressful. More recently, fully asleep DBS procedure has been brought to the field [42] but is yet to be democratized since it implies pre-surgical high-resolution imaging and intraoperative control using—if not magnetic resonance imaging (MRI)—at least a CT scan [47].

The role of hypnosis and hypnosedation (hypnosis with the adjunct of a sedative drug) during surgical procedures has been discussed over the past 15 years [2, 19–21, 35, 36, 40, 50, 54, 57] and both are currently practiced worldwide in various surgical fields such as thyroid surgery or gynecologic surgery, for example [48–50, 57]. Yet, the clinical potential of hypnosis in pain modulation is now well established [20, 21, 36, 40]; in a recent review including 49 studies and 135 patients, Fernandez *et* al. showed that hypnosis globally reduces the sympathetic responses and/or increases parasympathetic tone [22], eventually leading to reduced perioperative stress.

While the successful use of hypnosedation during awake neurosurgical procedures has been reported [4], the role of hypnosis during stereotactic procedures, in particular during SF mounting, has not yet been explored, mainly because brain mechanisms of hypnosis are poorly known and the hypnotizability varies among patients. Hence, the impact of hypnosis on the daily medical practice is probably reduced and the use of hypnosis may be seen as somewhat mysterious among the scientific community. Still, imaging studies and solid fundamental data are available in the literature [7, 11–13, 31, 58].

Another reason for the lack of data is the difficulty to set up a solid, blinded methodology aiming to determine the clinical, quantitative role of hypnosis.

In this context and in the perspective to improve our patients’ comfort and adherence to their treatment, our aim was to assess the role of hypnosis as an adjunct to LA to decrease pain and distress during the SF fixation in PD patients undergoing awake bilateral subthalamic nucleus (STN) DBS. Our hypothesis was that the adjunct of hypnosis to LA would reduce the overall procedural pain, anxiety, and perceived stress.

## Methods

### Generalities

This was a randomized, unblinded study designed with the collaboration of our epidemiology unit. Two groups were established: the hypnosis and the control group. We decided not to blind the study for both investigators and participants because it seemed unethical to suggest a fake hypnosis session to patients included in the control group. Therefore, patients knew their treatment allocation prior to the surgery. This certainly introduces a suggestibility bias, the caregiver being susceptible to transfer his/her expectations to the patient.

The study was registered on ClinicalTrials.gov (NCT 03074422) and was approved by the local ethics committee (IRB approval 2016–01843). Informed consent was obtained for all the patients included in the study.

**Fig. 1:**
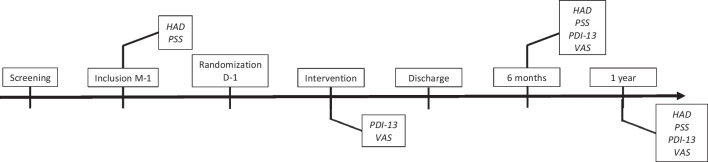
Summary of screening, inclusion, intervention, and follow-up milestones of the study. Patients suffering from Parkinson’s disease were screened during the regular multidisciplinary assessment for deep brain stimulation involving neurologists and neurosurgeons. Study inclusion was achieved 1 month before surgery. Patients were unblindedly randomized into the hypnosis or into the control group the day prior to the surgery. Procedural pain and distress were assessed after the mounting of the stereotaxic frame as well as at 6- and 12-month follow-up, while anxiety and stress were measured before the surgery as well as at 6- and 12-month follow-up. PD, Parkinson’s disease; DBS, deep brain stimulation; STN, subthalamic nucleus; M, month; HAD, hospital anxiety and depression scale; PSS, perceived stress scale; VAS, visual analogue scale; PDI-13, peritraumatic distress inventory-13 items; PCLS, post-traumatic stress disorder checklist state

Milestones and related assessments/outcomes are summarized in Fig. 1.

### Patients’ inclusion

A total of *N* = 19 patients with a diagnosis of PD based on the UK Parkinson’s Disease Society Brain Bank Criteria [28] were prospectively and consecutively included in the study (*N* = 13 males, mean age 63 years (43–73 years) (Table 1). All patients underwent STN DBS at our center. *N* = 10 patients were allocated to the hypnosis group and *N* = 9 patients were allocated to the control group. All patients had a diagnosis of PD. Aside from their allocation, cohorts were similar in terms of gender and age. The study started in January 2016 and the last patient was recruited in December 2021. Including the last FU visit, the study ran from January 2016 to December 2022.

**Table 1:** Baseline demographic data of the study

	Hypnosis (*n* = 10)	Control (*n* = 9)	*p*-value
Baseline demographics			
Age	63.8 (51–73)	61.7 (43–72)	-
Sex	5 M/5 F	8 M/1 F	0.06

One month prior to surgery, the study was presented to the patient by the surgeons (MVC and SM). Once enrolled, patients underwent a comprehensive pre-operative neuropsychological assessment performed by a senior neuropsychologist (SCC) specialized in the management of patients suffering from PD. The neuropsychological assessment included the French version of the perceived stress scale (PSS) 10 [4] and the French version of the hospital anxiety and depression scale (HADS) [63].

Inclusion criteria were as follows: (1) patients > 18 years, (2) with a diagnosis of PD based on the UK Parkinson’s Disease Society Brain Bank Criteria [28], (3) undergoing STN DBS, and (4) able to consent. Patients undergoing DBS for other indications than PD and/or in other targets than STN were excluded from the study after the screening, as well as patients suffering from moderate-to-severe psychiatric comorbidity.

### Workflow and randomization

The day prior to surgery, patients were admitted to our Neurosurgery Department. All participants had a pre-surgical consultation with our senior anesthesiologist (AW) and all patients were prepared for hypnosis. Our senior anesthesiologist is a board-certified trained professional with a national diploma in hypnotherapy and is also the director of the hospital center for hypnosis. The evening prior surgery, patients were randomly assigned to either the interventional (hypnosis and local anesthesia) or non-interventional (local anesthesia only) groups, using the blocked randomization, which was previously set by the epidemiology unit of the Geneva University Hospitals (Unité d’appui méthodologique, Centre de Recherche Clinique, Hôpitaux Universitaires de Genève) by the mean of sealed envelopes containing a paper on which the treatment allocation (interventional versus non-interventional group) was detailed. Treatment allocation was communicated to the team and to the patient immediately after the opening of the envelope.

All patients had their head shaved the evening before the surgery.

**Fig. 2:**
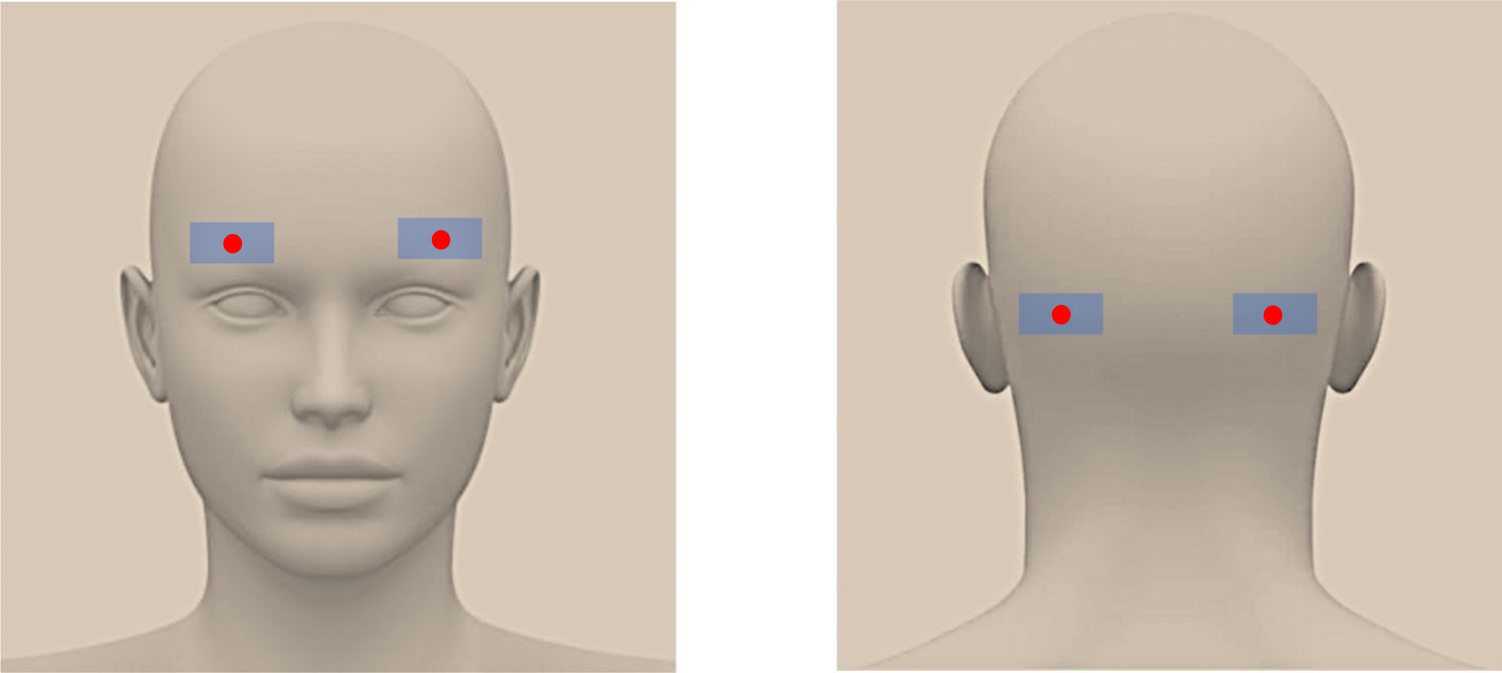
Forty-five min prior to frame mounting, four patches of prilocaïne/lidocaïne (5%) were applied to the skin of the patient, at the level of the fixation points of the Leksell headframe

The morning of the surgery, all patients had four patches of lidocaine/prilocaine 5% (EMLA patch 5%, Aspen Pharma Schweiz GmbH, Baar, Switzerland) applied to the skin in the frontal and occipital region at 06:45 a.m. (Fig. 2). The patches were left in place for 45 min. A Leksell SF Model G was used in all procedures (Leksell, Elekta, Stockholm, Sweden). Withdrawal of antiparkinsonian drugs was undertaken 12 h prior to surgery. No patients involved in the study had apomorphine pumps.

### Sample size

It was estimated a priori that the standard deviation for the pain score was 2 in the study population. To detect a difference of 3 between the mean of pain scores between the intervention and the control groups with a 90% power and a 5% α-risk, a sample of *N* = 22 patients with *N* = 11 patients in each arm was required. Unfortunately, the local sanitary regulation authorities stopped the local functional neurosurgery program in 2021. This was due to government-driven national reorganization of the functional program, resulting in *N* = 19 patients included in the study.

### Interventional group

The hypnotic session took place in three distinct phases: (1) phase of induction of the hypnotic state, (2) working phase, and (3) return to critical consciousness, as described elsewhere [27, 55]. All the sessions were conducted by the anesthesiologist/hypnotherapist (AW). To prepare the session, patients were asked to evoke personal resources and pleasant contextual memories with the hypnotherapist the evening prior to the surgery. These were used during hypnosis inducer thereafter.

**Fig. 3:**
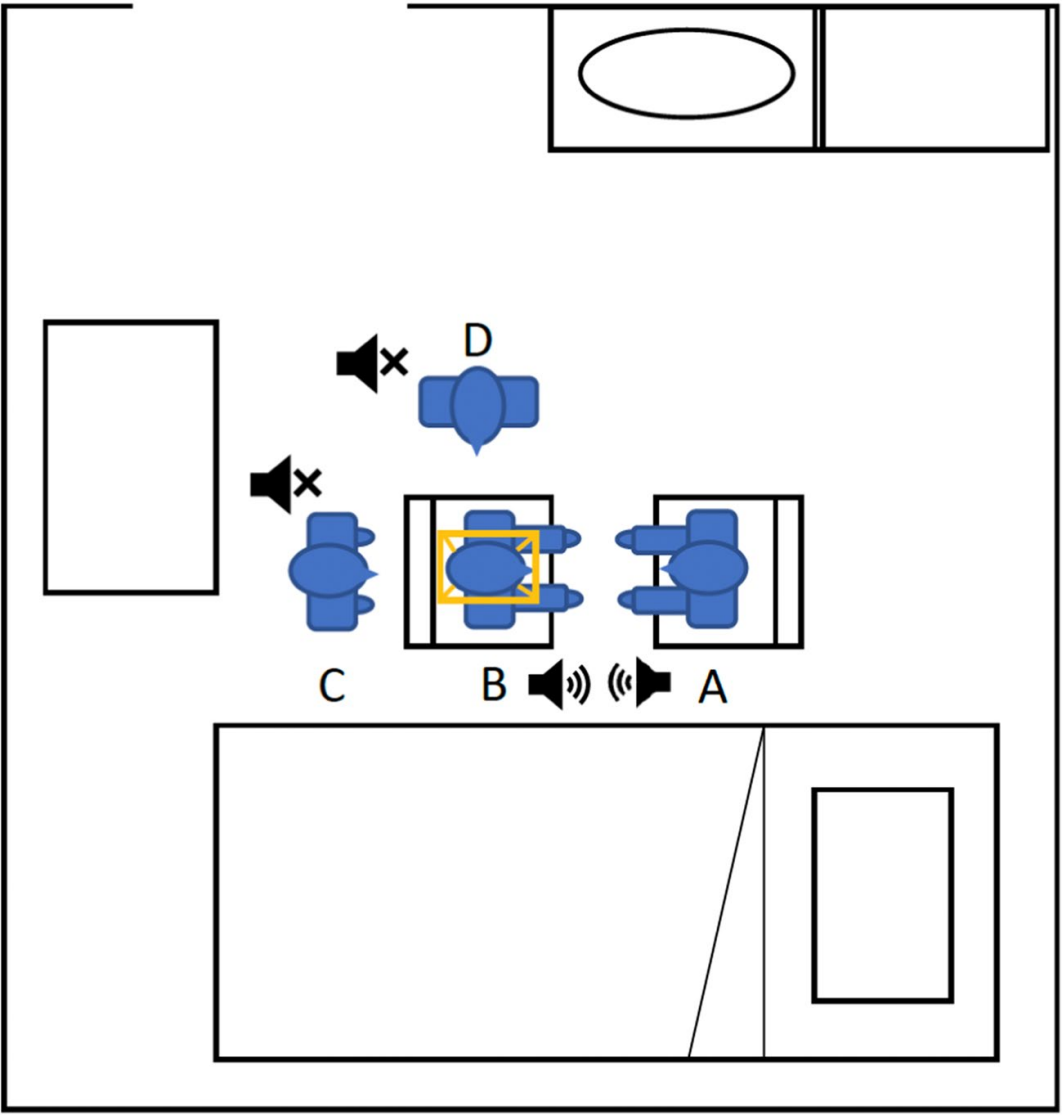
Typical setup during a hypnosis session prior to a deep brain stimulation surgery for Parkinson’s disease. The hypnotherapist (**A**) faces the patient (**B**). Behind the patient, one surgeon (**C**) mounts the stereotaxic frame. The other surgeon (**D**) stands on the side of the patient and holds the frame, ensuring that the head is straight. During the session, surgeons do not speak while the patient and the hypnotherapist communicate

The session was performed in a single-bed quiet room with the door closed. Usually, the patient was sitting on a chair, but occasionally, the procedure took place with the patient lying in bed in the presence of severe akinesia. All patients were OFF-medication during the hypnosis session. The hypnosis session was started on the day of the surgery at 07:15 a.m. in the patient’s room. The overall disposition of the room during the hypnosis session is shown in Fig. 3.

After circa 20 min, the working phase was reached, and the surgeons were allowed to enter the room. During the whole procedure, the surgeons were not allowed to talk directly to the patient. Whenever necessary, the surgeons had to talk to the anesthesist, who then transmitted the information to the patient. At first, the lidocaine/prilocaine patches (EMLA patch 5%, Aspen Pharma Schweiz GmbH, Baar, Switzerland)were removed and the skin was disinfected. A LA (rapidocaïn 200 mg/20 ml, in-house preparation) was performed at the sites where the pins would be inserted thereafter (Fig. 2). After 5 min, the headframe was fixed using four sharp pins which were screwed through the skin to the skull bone, sequentially. Once the procedure was completed, the patient was asked by the hypnotherapist to open his/her eyes and to return to consciousness. The hypnosis session was over. Overall, it lasted 30–40 min.

### Control group

In the control group, the mounting of the SF was performed in a single-bed quiet room with the door closed. The patient was usually sitting on a chair, but occasionally, the procedure took place with the patient lying on the bed. The procedure usually started at 07:30 a.m. At first, the rapidocaïn/prilocaïn patches (EMLA patch 5%, Aspen Pharma Schweiz GmbH, Baar, Switzerland) were removed and the skin was disinfected. A LA was performed at the site where the pins would be inserted (rapidocaïn 200 mg/20 ml, in-house preparation). After 5 min, the headframe was fixed using four sharp pins which were screwed through the skin, sequentially. All patients included in the control group were OFF-medication during the mounting of the SF.

### Surgical procedure

After the mounting of the SF, the patient was transferred to the radiology department where a cerebral CT scan was performed and fused with the pre-operative planning images. Thereafter, the patient was transferred to the operating room (OR).

The patient was positioned supine, with an oxygen mask in place. Antibiotic prophylaxis was achieved using intravenous cefazolin (Céfazoline Sandoz, Sandoz Pharmaceuticals AG, Rotkreutz, Switzerland). Mild sedation was achieved during patient preparation using intravenous propofol (Propofol-Lipuro 1% 1 g/100 ml, B Braun Medical, Sempach, Switzerland). Local scalp anesthesia was performed using 10 cc rapidocaïn (rapidocaïn 200 mg/20 ml, in-house preparation) for each incision. Two arciform incisions were then performed under analgo-sedation and careful hemostasis was achieved. Two burr holes were drilled. Thereafter, the sedation was stopped to obtain full collaboration of the patient during micro-electrode recording and neurological testing. Once the definitive position of the electrode was decided, a definitive electrode was positioned under X-ray control (Boston Scientific Cartesia, Boston Scientific, MA, USA). Once both definitive electrodes were in place, the skin was closed using subcutaneous sutures and staples.

### Assessment of outcomes

The study design is presented in Fig. 1.

#### Baseline and interventional outcomes

##### Primary outcome

The primary outcome was the intensity of pain perceived during the procedure, as measured by the visual analogue scale (VAS). Directly after SF fixation, patients were asked to report the mean (VAS_mean_) and maximal (VAS_max_) procedural pain.

##### Secondary outcomes

Baseline stress, anxiety, and depression were assessed using the perceived stress scale (PSS) and hospital anxiety and depression scale (HADS and their anxiety (HADSa) and depression (HADSd) subscales), respectively. The procedural distress was assessed right after the procedure by the neuropsychologist (SCC) using the peritraumatic distress inventory (PDI-13) [30].

##### Post-interventional outcomes and follow-up

Follow-up (FU) assessments were performed at 6-month and 1-year (FU). Peritraumatic distress as well as anxiety and depression were measured during the FU period. To do so, PSS, PDI-13, and HADS questionnaires were completed by our senior neuropsychologist (SCC) during a phone call or during a clinical FU visit.

**Fig. 4:**
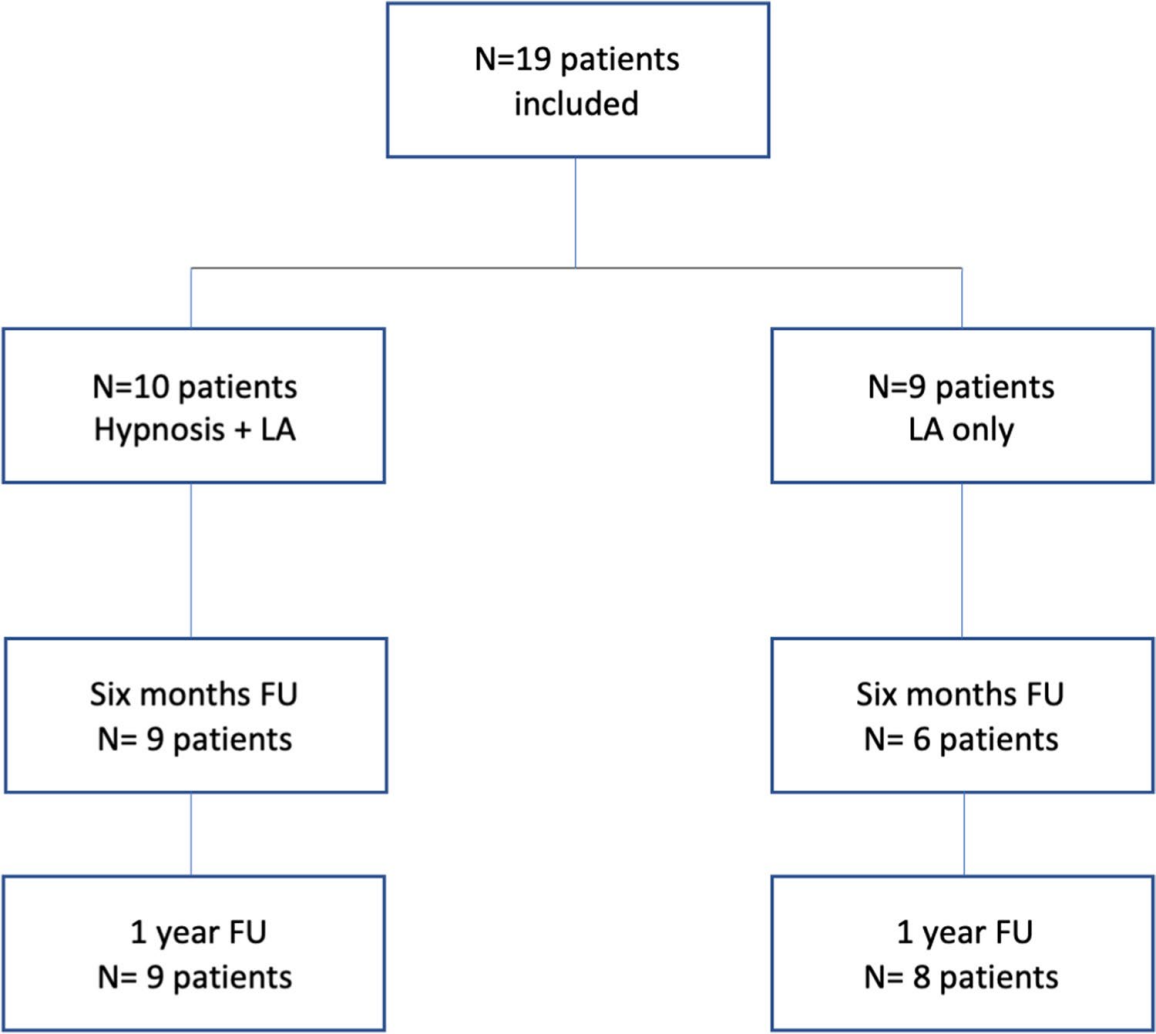
Study flowchart summarizing randomization in both groups and further distribution of the study population throughout the study period. There were no cross-overs. One patient was lost of follow-up in the intervention group, while three patients did not attend the 6-month assessments and one patient did not respond to the 1-year questionnaires

There were neither cross-overs nor withdrawals. *N* = 1 patient in the intervention group was lost of FU, while *N* = 3 patients and *N* = 1 patient in the control group were lost of FU at 6- and 12-month FU, respectively (Fig. 4).

### Statistical analysis

The statistical analysis was performed using GraphPad PRISM version 9.3.1 (GraphPad Software, LLC, San Diego, CA (USA)). The significant *p*-value was defined at 0.05. Unpaired *t*-tests were used to compare groups and multiple paired *t*-tests were used to compare the evolution of the mean scores over FU.

### Adverse events

There were no adverse events during the study period.

### Placebo and nocebo effects

Due to the unblinded design of the study, patients included in the interventional group were able to benefit from a placebo effect. Correspondingly, the consciousness among the patients in the control group of not receiving additional hypnosis treatment could have generated a nocebo effect. This is discussed further in the limitation section.

## Results

### Pre-operative measures

Pertaining to their pre-operative HADS, patients in the hypnosis group had similar HADSd and HADSa scores (6.2 ± 4.3 versus 6.7 ± 1.92, 95% CI [− 3.896 to 2.740], *p* = 0.72 and 6.7 ± 4.2 versus 7.7 ± 3, 95% CI [− 4.549 to 2.615], *p* = 0.58 in the hypnosis and control groups, respectively). Regarding the pre-operative PSS scores, both groups were similar (26.1 ± 6.3 versus 25.1 ± 7, 95% CI [− 5.440 to 7.418], *p* = 0.75). Pre-operative data are summarized in Table 2.

**Table 2:** Study timeline, procedures, and assessment at screening, during intervention, and follow-up. *HAD* hospital anxiety and depression scale, *PSS* perceived stress scale, *PDI-13* peritraumatic distress inventory, *VAS* visual analogue score, *PCLS* post-traumatic stress disorder checklist state

	Hypnosis (*n* = 10)	Control (*n* = 9)	*p*-value
Pre-operative			
HADd	6.2 ± 4.3	6.7 ± 1.92	0.72
HADa	6.7 ± 4.2	7.7 ± 3	0.58
PSS	26.1 ± 6.3	25.1 ± 7	0.75
After procedure			
VAS_mean_	5.6 ± 2.1	6.4 ± 1.2	0.31
VAS_max_	7.6 ± 2.1	8.6 ± 1.6	0.28
PDI-13	7.1 ± 4	9.6 ± 4.7	0.23
6-month FU			
VAS_mean_	5.3 ± 3.4	6.2 ± 2.5	0.7
VAS_max_	6.2 ± 3.4	8.2 ± 1.8	0.37
PDI-13	6.7 ± 8.8	11.2 ± 8.9	0.40
PCL-S	24.2 ± 6.1	26.2 ± 11.7	0.7
PSS	25.6 ± 9.4	26.2 ± 6	0.94
HADd	5.9 ± 3.6	8.5 ± 3.9	0.25
HADa	6.4 ± 1.9	6 ± 3.3	0.59
1-year FU			
VAS_mean_	4.9 ± 2.8	6.3 ± 2	0.26
VAS_max_	7.8 ± 2.3	8.1 ± 2.1	0.58
PDI-13	10.8 ± 9.8	14.8 ± 12.3	0.35
PCL-S	21.2 ± 4.7	25.6 ± 12.2	0.34
PSS	23.2 ± 9	27.4 ± 5.2	0.41
HADd	5.2 ± 3.8	7.5 ± 4.5	0.63
HADa	7.2 ± 3.4	8.5 ± 3.3	0.94

### Pain during procedure

Pertaining to the pain experienced during the procedure, there was no difference between both groups during SF fixation. In the hypnosis group, VAS_mean_ during the procedure was 5.6 ± 2.1, versus 6.4 ± 1.2 in the control group (95% CI [− 2.550 to 0.8611], *p* = 0.31). Pertaining to the VAS_max_ reported during the procedure, the intervention group reported a mean score of 7.6 ± 2.1 versus 8.6 ± 1.6 (95% CI [− 2.786 to 0.8750], *p* = 0.28). Data on pain reported during the procedure are summarized in Table 2.

### Follow-up data

**Fig. 5:**
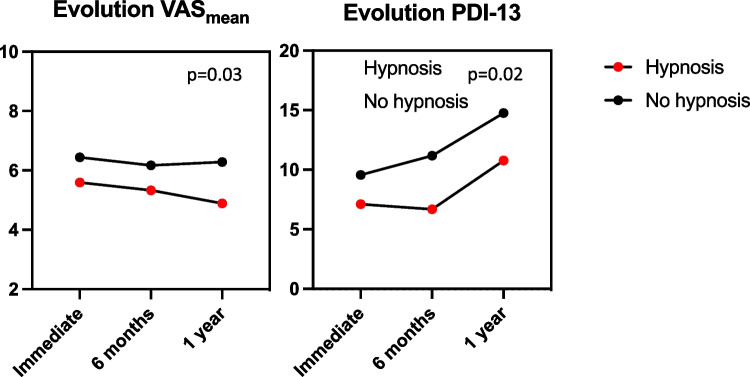
Evolutions of VAS_mean_ (left) and PDI-13 (right) scores in both cohorts throughout the study period. Regarding VAS_mean_, both cohorts showed an overall reduction of pain quotation during follow-up, which was more there was a pattern of reduction of scores in both cohorts, which was significantly more marked in patients who received hypnosis. Regarding PDI-13 scores, both cohorts showed increased scores after the procedure, which was significantly higher in the control group. VAS, visual analogue scale; PDI, peritraumatic distress inventory

Data on stress, anxiety, and pain at 6-month and 1-year FU are shown in Table 2. While there was no statistical difference in terms of VAS, HADS, and PDI-13 scores during FU between both cohorts, the pattern of evolution significantly differs pertaining to VAS_mean_ (*R*^2^ = 0.93, 95% CI [0.2245 to 1.825], *p* = 0.03) and PDI-13 scores (*R*^2^ = 0.94, 95% CI [1.006 to 6.279], *p* = 0.02; Fig. 5).

## Discussion

### Overall findings

We compared the efficacy of hypnosis in reducing acute pain during SF mounting in patients suffering from PD undergoing awake bilateral STN-DBS. While there is no absolute difference between intervention and control groups in terms of perceived pain, anxiety, and perioperative distress, the two arms showed a significantly different evolution in terms of pain recalling and peritraumatic memories, as measured by VAS and PDI-13 scores. To our knowledge, this is the first prospective, randomized study to assess the role of hypnosis in patients undergoing frame fixation for STN DBS in PD. Even though this study is flawed by several limitations, it provides insights on the optimization of pain management during a painful procedure in PD patients using hypnosis as an adjunct to LA.

Since the early 1990s, several research groups identified the existence of hypnosis-related phenomena and their influence on the pain signal perception, showing that there is a modulation of the anterior cingulate area activity together with modified interconnectivity with other critical regions involved in nociception [20]. From then, the potential of hypnosis in pain modulation and, more extensively, perioperative patient management was established [20, 21, 36, 40], as hypnosis has been used even during the resection of brain tumors [19, 50, 57, 62]. However, there is room for improvement in our understanding of hypnosis, its development, and its implementation into the daily clinical practice [31].

Pain during SF fixation in awake DBS has long been a concern for surgeons and patients. In 2001, Watson *et* al. compared supraorbital and greater occipital nerve blocks with subcutaneous infiltration during SF fixation in patients suffering from PD, using VAS (0–100) to quantify the procedural pain [59]. Neither technique was superior in preventing pain associated with pin placement at the frontal or occipital sites and the procedure was reported to be globally painful by patients. However, nerve blocks were less painful than subcutaneous LA at both the frontal (VAS, 34 ± 24 vs 49 ± 25) and occipital (VAS, 34 ± 21 vs 49 ± 23) sites. Aside from pain-reduction procedures, frameless stereotactic interventions [24, 53] have been promoted recently, as have been fully asleep DBS surgeries [6, 9, 18, 32]

Since not only SF fixation but also the skin incision are painful, the scalp block, where all areas of the frame and the sites of the skin incisions are anesthetized, can be performed. This results in patient comfort improvement and saves on analgesics as well as antihypertensive drugs, as shown by Krauss *et* al. [34]. In their study, the authors were able to show that patients who had scalp block showed lower mean systolic blood pressure and heart rate compared with patients who had LA. Thereafter, more antihypertensives were required to achieve blood pressure control in the LA cohort. As previously stated, hypnosis is also effective in reducing the sympathetic tone as well as increasing the parasympathetic perioperative response [22].

Along with these findings, Schnur *et* al. showed the use of hypnosis to reduce emotional distress associated with medical procedures, underlying the need of hypnosis and its relevance when it comes to management during invasive procedures, on the basis of 26 trials including 2342 participant articles [46].

### Hypnosis and acute pain

According to the American Psychological Association, hypnosis is defined as “a state of consciousness involving focused attention and reduced peripheral awareness characterized by an enhanced capacity for response to suggestion” [17]. The effectiveness and reliability of hypnosis in acute pain disorders and procedural pain have been previously assessed elsewhere [1, 5, 8, 14–16, 20, 33, 37, 41, 44, 51, 52, 54, 60].

Pain is associated with modulation of the activation of the primary and secondary somatosensory areas, the insula, and the anterior cingulate cortex. In addition, individual characteristics and the context play a role in pain modulation [29, 38]. In our study, the procedural pain reported in the two groups was similar during the procedure, at 6-month and 1-year FU. This is probably due to the procedure itself, where four sharp pins are directly inserted through the skin, and pressure is applied by screwing them into the skull.

### Pain and peritraumatic distress recall

The recall of the procedure, in the perspective of pain and peritraumatic distress, significantly differs between intervention and control groups, showing that patients who received hypnosis tend to report lower pain and distress scores over time. Due to the study protocol, the FU was limited to 1 year, but a longer FU could be useful to confirm the tendency towards minimization of PDI-13 and VAS scores in the hypnosis group. This phenomenon can be explained by the previously reported reduced activation in the left amygdala and bilaterally in the anterior cingulate cortex (ACC), insula, and hippocampus during hypnosis [26].

Altogether, our data support the beneficial role of hypnosis in reducing unpleasant and stressful memories after highly painful procedures. In that sense, hypnosis could possibly play a role in long-term memory of pain levels, which tends to decrease with time. In this perspective, hypnosis appears to modulate the memory of pain by making it less prominent over time.

### Local versus general anesthesia

The mounting of the SF on the patient’s head is performed under LA, as the patient is awake during the mounting procedure and later on during the surgical intervention. This moment is reported as “painful” to “extremely painful” by patients, and most of them confess to keep a very unpleasant memory of the event, even several years after the procedure and despite the fact that the surgery had a positive effect on their functional outcome.

We keep the patient awake because the procedure requires fully collaboration as the head must be maintained in a neutral position. Furthermore, awake mounting of the SF reduces the risks of procedure-related adverse events and avoids orotracheal intubation, which also carries its own risks. Finally, it allows to keep the patient awake during the subsequent microelectrode recording and testing of the electrode during the DBS procedure itself. Alternatively, fully asleep DBS is increasingly advocated as a standard of care but requires intra-operative imaging, which is far from being democratized worldwide yet.

### Parkinsonians as a specific subgroup of patients experiencing pain

Alteration of pain perception, as a non-motor symptom resulting from abnormal processing of the sensory input through the basal ganglia, is part PD spectrum [10, 25]. It has been previously reported that patients with PD had a lower pain threshold compared to non-PD age-adjusted patients; the duration and severity of PD were directly correlated to the reduction of the threshold [39]. These results were corroborated by Zambito *et* al. [61].

On the contrary, other groups advocate hypoalgesia in patients suffering PD: Tycocki *et* al. compared subjective pain intensity during stereotactic frame fixation in patients undergoing STN DBS for PD to the pain experienced in non-parkinsonian patients undergoing frame fixation for stereotactic brain biopsy. The authors were able to report that patients with PD had overall lower VAS scores than non-PD patients [56].

### Perspectives

Aside from the debate between the fully asleep DBS procedures with direct targeting and microelectrode recording-guided awake DBS procedures, the fixation of the steSF is an issue, when it comes to awake patients, because of the pain and the resulting anxiety and distress. Our results clearly indicate that there is an increase of PSS, PDI-13, and HADS scores from baseline to the end of the FU. Even though these results are not statistically significant and are flawed with methodological limitations resulting from unblinding for treatment, there may be an impact of the procedure on the patient, which could be demonstrated in larger cohorts. Since we focused our endpoint on pain, the study was not designed to include more patients.

The lack of blinding may have biased patients in both groups: it is possible that the favorable outcome in the interventional group with respect to the evolution of the VAS_mean_ could depend on a placebo effect generated by the patients’ consciousness of having been allocated to the hypnosis group. It is also possible that the health care professionals could unconsciously and involuntarily transfer their expectations of a better outcome on pain in the interventional group compared to the control group. Alternatively, the consciousness among patients in the control group of not receiving additional hypnosis may have generated a nocebo effect, with negative expectation concerning the outcome on pain intensity. However, the absence of difference in every measured outcome may suggest that these effects were not predominant. Aside, the different evolution of pain memory over time may indicate that there is, in fact, a role played by the hypnosis itself.

## Strengths and limitations

Data were prospectively acquired by a certified study nurse. Patients were systematically assessed by a board-certified neuropsychologist with a specific board certification for hypnosis. The pre-operative and perioperative patient pathway was well established, ensuring a smooth and fluid throughout the hospital stay.

Our study suffers from several drawbacks, i.e., its design, the limited amount of data acquired, and the study population itself. The unblinding is due to the hypnosis procedure itself, as the patient *actually* knows whether s/he is receiving hypnosis or not. A potential solution to overcome this critical issue would be to run a fake hypnosis session by an unexperienced professional. This would in turn raise an ethical question. Alternatively, placebo and nocebo effects were probably generated by the study design, since healthcare professionals involved in the study could transfer their expectations to the patient, who in turn might have felt positive or negative feelings related to what they may have perceived as a good or improper treatment (whether hypnosis or not).

Only *N* = 19 patients were included as local sanitary regulations stopped our functional neurosurgery program, while a sample of *N* = 22 patients was required. However, the very similar outcomes on perceived pain do not suggest that three additional patients would have led to significantly different results. *N* = 2 patients were lost of FU at 1 year due to moving abroad (*N* = 1) and other disabling medical conditions (*N* = 1). Lastly, as previously stated, PD patients may have a distorted perception of the hypnosis session, especially since they were OFF-med during the procedure.

Altogether, these limitations somewhat restrict the representativity of our cohort and limit the generalizability of our results. However, since no clear-cut result in favor of a cohort over the other was found, it may be inferred that the role of these biases may be limited.

Lastly, we did not measure the quality of the hypnotic state because there is no reliable, applicable, and reproducible specific score of the hypnosis state, underlining the need for an objective, clinical score of hypnosis.

## Conclusion

Hypnosis does not seem to influence perceived pain, anxiety, and distress during the awake stereotactic frame fixation procedure. However, the results suggest that it might possibly modulate pain memory over time and may prevent the integration of awake painful procedures as a bad experience into the autobiographical memory of patients suffering from PD. Larger cohorts included in randomized controlled studies are necessary to confirm their results.

## Data Availability

Data and material are available upon request.

## Abbreviations

ACC: Anterior cingulate cortex
DBS: Deep brain stimulation
FU: Follow-up
IRB: Institutional review board
HADS: Hospital anxiety and depression scale
HADSa: Hospital anxiety and depression scale, anxiety subscale
HADSd: Hospital anxiety and depression scale, depression subscale
LA: Local anesthesia
OR: Operating room
PD: Parkinson’s disease
PDI-13: Peritraumatic distress inventory
PSS: Perceived stress scale
SF: Stereotactic frame
STN: Subthalamic nucleus
VAS: Visual analogue scale

## Declarations

### Study registration and protocol

The study protocol is available upon request and on https://clinicaltrials.gov/ct2/show/NCT03074422.

Study number 2016–01843 (Commission Cantonale d’Ethique de la Recherche).

Clinicaltrials.gov identifier: NCT03074422.
